# Diagnostic performance of MRI and transvaginal ultrasound for urinary tract endometriosis: a systematic review and meta-analysis

**DOI:** 10.1007/s00404-026-08553-6

**Published:** 2026-08-10

**Authors:** Anna Pitsillidi, Laura Vona, Konstantinos Kyriakoulis, Panagiotis Iliakis, Guglielmo Stabile, Günter Noé

**Affiliations:** 1 Department of OB/GYN, Rheinland Klinikum Neuss, Preußenstrasse 84, 41464 Neuss, Germany; 2 Department of Medical and Surgical Sciences, Institute of Obstetrics and Gynaecology, University of Foggia, 71122 Foggia, Italy; 3 First University Department of Cardiology, National and Kapodistrian University of Athens, Athens, Greece; 4 Department of OB/GYN, University of Witten Herdecke, 58448 Witten, Germany

**Keywords:** Endometriosis, Ureter, Bladder, Ultrasound, MRI

## Abstract

**Objective:**

To compare the diagnostic accuracy of transvaginal ultrasound (TVUS) and magnetic resonance imaging (MRI) for detecting urinary tract endometriosis (UTE), including ureteral and bladder involvement.

**Methods:**

A systematic review and meta-analysis were conducted according to PRISMA 2020 guidelines. PubMed, Embase, and Scopus were searched from inception to December 2025 for diagnostic accuracy studies evaluating TVUS and/or MRI for UTE. Surgical findings, with or without histopathological confirmation, were used as the reference standard. Studies reporting sufficient data to construct 2 × 2 contingency tables were included. Pooled sensitivity and specificity with 95% confidence intervals (CI) were calculated using a bivariate random-effects model, with separate analyses for ureteral and bladder endometriosis.

**Results:**

Thirty-six studies met the inclusion criteria. For ureteral endometriosis, TVUS demonstrated a pooled sensitivity of 0.89 (95% CI 0.65–0.97) and specificity of 1.00 (95% CI 0.97–1.00), while MRI showed a pooled sensitivity of 0.96 (95% CI 0.50–1.00) and specificity of 1.00 (95% CI 0.96–1.00). For bladder endometriosis, TVUS yielded a pooled sensitivity of 0.70 (95% CI 0.55–0.81) and specificity of 1.00 (95% CI 0.99–1.00), whereas MRI demonstrated a pooled sensitivity of 0.77 (95% CI 0.58–0.89) and specificity of 0.99 (95% CI 0.98–1.00). Substantial heterogeneity was observed, particularly for sensitivity estimates.

**Conclusion:**

TVUS and MRI show excellent specificity for diagnosing urinary tract endometriosis. MRI provides slightly higher sensitivity for ureteral disease, while both modalities demonstrate moderate sensitivity for bladder involvement. A complementary, site-specific imaging approach is, therefore, supported, with cautious interpretation of negative findings.

**Supplementary Information:**

The online version contains supplementary material available at https://doi.org/10.1007/s00404-026-08553-6.

## Introduction

Endometriosis is a chronic inflammatory condition affecting approximately 10% of women of reproductive age and is characterized by the presence of endometrial-like tissue outside the uterine cavity. Deep infiltrating endometriosis (DIE), defined as infiltration of endometriotic tissue more than 5 mm beneath the peritoneal surface, represents a severe phenotype associated with significant morbidity, complex surgical management, and impaired quality of life [1, 2].

Urinary tract endometriosis (UTE) is an uncommon, but clinically significant manifestation of DIE, occurring in approximately 1–6% of women with endometriosis and up to 50% of those with deep infiltrating disease [3, 4]. The bladder is the most frequently involved site, followed by the ureters. While bladder endometriosis may present with cyclic urinary symptoms, ureteral involvement is often asymptomatic and may lead to silent hydronephrosis and irreversible loss of renal function if not promptly diagnosed [5]. Accurate preoperative identification of urinary tract involvement is, therefore, essential for appropriate surgical planning and multidisciplinary management. Imaging plays a central role in the diagnosis and mapping of urinary tract endometriosis. Transvaginal ultrasound (TVUS) is widely used as a first-line modality due to its accessibility, cost-effectiveness, and high spatial resolution when performed by experienced operators [6]. In expert hands, TVUS allows dynamic assessment of pelvic structures and has demonstrated good diagnostic performance for several manifestations of DIE [7, 8]. However, TVUS is inherently limited to evaluation of the pelvic portion of the ureter; consequently, assessment of hydronephrosis requires abdominal ultrasonography rather than reliance on transvaginal examination alone [6]. Magnetic resonance imaging (MRI), on the other hand, offers excellent soft-tissue contrast and a comprehensive anatomical overview, which may be advantageous in assessing lesion extent, multifocal disease, and ureteral involvement [9].

Despite the widespread use of both imaging modalities, uncertainty remains regarding their relative diagnostic accuracy for UTE. Reported estimates of sensitivity and specificity vary considerably across studies, reflecting heterogeneity in study design, patient populations, imaging protocols, operator expertise, and reference standards. Moreover, urinary tract involvement—particularly ureteral disease—often remains underdiagnosed, further complicating accurate assessment of imaging performance.

Although previous meta-analyses have evaluated the diagnostic accuracy of TVUS and MRI for DIE involving the urinary tract, new primary studies have since been published, and important methodological differences exist among available reviews. These include variation in inclusion criteria, disease definitions, and analytical approaches, as well as limited differentiation between bladder and ureteral involvement in some analyses. Consequently, uncertainty persists regarding the optimal imaging modality for preoperative assessment of urinary tract endometriosis.

Therefore, the aim of this systematic review and meta-analysis is to provide an updated and comprehensive comparison of the diagnostic accuracy of TVUS and MRI for the detection of UTE, including both bladder and ureteral disease. By synthesizing the most recent evidence using a standardized methodological approach, this study seeks to inform clinical decision-making and support optimal imaging strategies in women with suspected UTE.

## Material and methods

### Protocol and registration

This systematic review and meta-analysis were conducted in accordance with the preferred reporting items for systematic reviews and meta-analyses (PRISMA) 2020 guidelines [10]. The review protocol was registered in the PROSPERO database (registration number: CRD420251275906).

### Eligibility criteria

Studies were eligible for inclusion if they were prospective or retrospective diagnostic accuracy studies evaluating TVUS and/or MRI as index tests for the detection of UTE involving the ureter and/or urinary bladder. Eligible populations included women with suspected endometriosis who subsequently underwent surgical exploration by laparoscopy or laparotomy, with or without histopathological confirmation, which served as the reference standard.

Studies assessing both ureteral and bladder endometriosis were included only when diagnostic accuracy data were reported separately for each anatomical site. When both TVUS and MRI were evaluated within the same population, studies were included only if diagnostic accuracy results for each imaging modality were reported independently. To be eligible, studies were required to report sufficient data to construct 2 × 2 contingency tables (true positives, false positives, true negatives, and false negatives) or to provide data from which these values could be derived.

Case reports, small case series, narrative or systematic reviews, editorials, letters, conference abstracts, animal studies, and non-original articles were excluded. Studies were also excluded if they did not use surgical confirmation as the reference standard, failed to report diagnostic accuracy data separately for ureteral and bladder lesions, pooled results from TVUS and MRI without separate reporting, included overlapping populations without extractable independent data, or were not published as full-text articles in English.

### Information sources and search strategy

A comprehensive literature search was independently performed by two reviewers (L.V. and A.P.) using PubMed, Embase, and Scopus, from database inception to [all studies published up to December 2025], without restrictions on publication date. The search strategy combined controlled following keywords and MeSH terms related to endometriosis, urinary tract, bladder, ureter, transvaginal ultrasound, and magnetic resonance imaging. Boolean operators (AND, OR) were used to combine search terms appropriately. The core search strategy applied was as follows: (endometriosis OR "urinary tract endometriosis") AND (bladder OR ureter) AND ("transvaginal ultrasound" OR TVUS) AND ("magnetic resonance imaging" OR MRI).

The complete search strategy, including all database-specific adaptations, is available in the PROSPERO protocol (CRD420251275906).

Reference lists of all included studies and relevant reviews were manually screened to identify additional eligible articles. When required, corresponding authors of primary studies were contacted to obtain clarification or missing data.

### Data items

For each included study, data were extracted using a standardized data collection form. Extracted variables included first author and year of publication, study design, country, clinical setting, and sample size. Baseline patient characteristics were recorded when available, including mean age and body mass index (BMI).

Diagnostic accuracy data for TVUS and MRI were extracted separately, including the number of true positives, false positives, true negatives, and false negatives for each modality. When both imaging techniques were evaluated within the same study, data were extracted separately for each test.

For studies assessing ureteral endometriosis, the unit of analysis (patient- or ureter-based) was defined according to the original study design. Diagnostic accuracy data were extracted according to the unit of analysis reported in each primary study. Information regarding the reference standard was extracted, including surgical confirmation by laparoscopy or laparotomy, with or without histopathological verification. When required, missing data were derived from published text, tables, or supplementary materials.

### Risk of bias assessment

The risk of bias and applicability concerns of the included studies were independently assessed by two reviewers (A.P. and P.I.) using the QUADAS-2 tool [11]. The domains evaluated included patient selection, index test, reference standard, and flow and timing. Any disagreements were resolved by consensus.

### Statistical analysis

Meta-analyses of diagnostic accuracy were performed separately for ureteral and bladder endometriosis, and TVUS and MRI. Pooled estimates of sensitivity and specificity, with corresponding 95% confidence intervals (CI), were calculated using a random-effects bivariate model for diagnostic test accuracy, which accounts for the correlation between sensitivity and specificity across studies. Respective positive (LR+) and negative likelihood ratios (LR-) were calculated. A sensitivity analysis was performed including studies that used both TVUS and MRI.

Forest plots were generated to display study-level and pooled estimates of sensitivity and specificity. Summary receiver operating characteristic (SROC) curves were constructed to illustrate overall diagnostic performance, including confidence and prediction regions.

Statistical heterogeneity was assessed qualitatively through visual inspection of forest plots and SROC curves. All analyses were performed using Stata, v16 (StataCorp LLC) statistical package. A two-sided *P* < 0.05 was considered statistically significant.

## Results

### Study selection

**Fig. 1:**
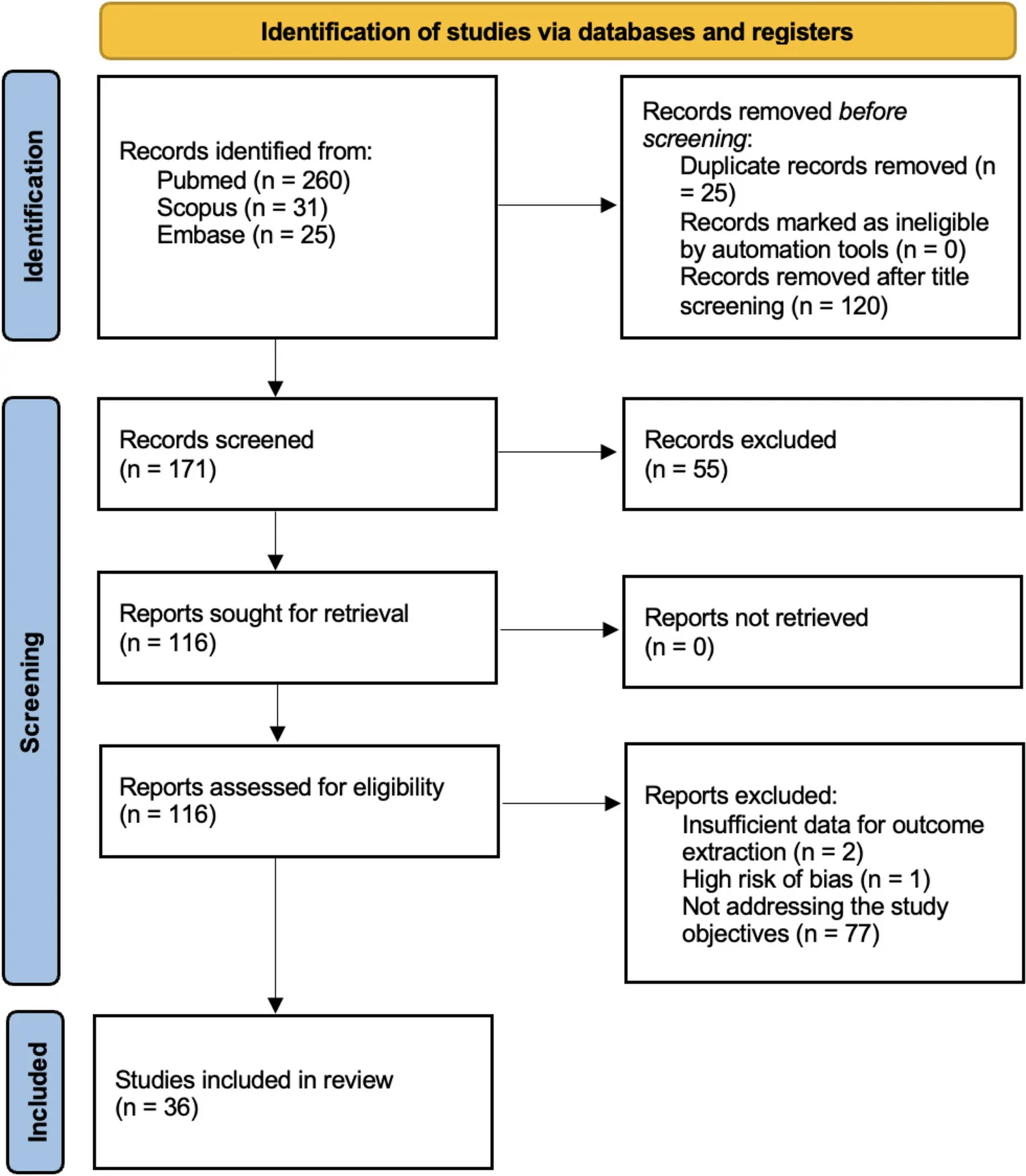
PRISMA flow diagram

The literature search identified 316 records through database searching, including PubMed (*n* = 260), Scopus (*n* = 31), and Embase (*n* = 25). After removal of duplicates (*n* = 25) and records excluded prior to screening for other reasons (*n* = 120), 171 records were screened by title and abstract. Of these, 55 records were excluded. A total of 116 full-text articles were assessed for eligibility. Following full-text review, 80 studies were excluded because they did not address the study objectives (*n* = 77), lacked sufficient data for outcome extraction (*n* = 2) [12, 13], or were judged to be at high risk of bias according to the QUADAS-2 assessment (*n* = 1), specifically the study by Balleyguier et al. [14]. Ultimately, 36 studies met the inclusion criteria and were included in the qualitative and quantitative synthesis (Fig. 1).

### Study characteristics

The included studies evaluated the diagnostic performance of TVUS and/or MRI for the detection of urinary tract endometriosis involving the bladder and/or ureters. Both prospective and retrospective study designs were represented, with sample sizes ranging from fewer than 50 to more than 300 participants.

Across all studies, surgical findings, with or without histopathological confirmation, were used as the reference standard. Most investigations were conducted in tertiary referral centres with expertise in endometriosis imaging. Reporting of patient characteristics, urinary symptoms, and hydronephrosis varied across studies. Detailed characteristics of patients with ureteral endometriosis are summarized in Table 1, while those patients with bladder endometriosis are provided in Table 2.

**Table 1:** Characteristics of patients with ureter endometriosis

Study	Type	Country	Setting	*N*	Age (mean ± SD)	BMI (kg/m^2^) (mean ± SD)	Symptoms (%)	Hydronephrosis (%)	Endometriosis (%)	Imaging protocol
Chamié 2009 [23]	RS	Brazil	SI	92	33.0		8 (0.09)		8 (9)	MRI: 1.5T; T2 + T1 FS; contrast; uro-MRI (selected cases)
Vimercati 2012 [24]	RS	Belgium	SI	90	34.0		16 (0.18)		8 (9)	TVUS; no bowel prep; dynamic assessment (sliding sign); ureteral involvement via hydroureter MRI: 1.5T; T2 + T1 FS; contrast; antispasmodic
Exacoustos 2014 [16]	RS	Italy	SI	208	35.6 ± 5.7		31 (0.15)		29 (14)	TVUS ± TRUS; no prep; partial bladder filling.
Sillou 2015 [18]	RS	France	MC	35	33.5		33 (0.94)	7 (0.20)	31 (89)	MRI: 1.5T; T2 + T1 FS; ± contrast; uro-MRI
Pateman 2015[17]	RS	England	SI	164	36.1 ± 7.8			10 (0.06)	13 (8)	TVUS; partially filled bladder; direct ureter assessment; + renal US
Hudelist 2018 [25]	PS	Austria, UK	MC	207	33.9 ± 5.2		20 (0.10)	23 (0.11)	23 (11)	TVUS ± transabdominal US
Alborzi 2017 [26]	PS	Iran	SI	317	31.0 ± 5.4		0 (0.00)		2 (1)	TVUS/TRUS; bowel prep; partial bladder filling MRI: 1.5T; T2 + T1 FS; contrast; partial bladder; vaginal gel
Zannoni 2017 [27]	PS	Italy	MC	94	37.0 ± 5.3	23.0 ± 3.7	13 (0.14)		31 (33)	TVUS; indirect ureter assessment; + transabdominal renal US
Carfagna 2018 [6]	PS	Italy	SI	200	34.7 ± 6.1	22.6 ± 4.8	0 (0.00)	6 (0.03)	13 (7)	TVUS; ureter visualized along length; partially filled bladder; renal US
Hernández 2019 [19]	RS	Spain	SI	48	34.0 ± 6.0	22.0 ± 2.7	10 (0.21)		6 (13)	TVUS; bowel prep; partially filled bladder MRI: 1.5T; T2 + T1 FS; bowel prep; bladder & ureters; rectal & vaginal gel
Indrielle-Kelly 2020 [28]	PS	Czech Republic	SI	49	32.4 ± 5.3	23.6 ± 3.9	8 (0.16)		5 (10)	TVUS + transabdominal US; ± bowel prep; partial/moderate bladder filling MRI: 1.5–3T; T2 + T1 ± FS; ± bowel prep; moderate bladder filling; rectal & vaginal gel; whole pelvis ± upper urinary tract
Bielen 2020 [29]	PS	Belgium	SI	74	32.0			3 (0.04)	29 (39)	TVUS ± transabdominal US; ± bowel prep; partial/moderate bladder filling MRI: 1.5T; T2 + T1 ± FS; ± bowel prep; moderate bladder filling; rectal & vaginal gel; whole pelvis ± upper urinary tract
Yin 2020 [30]	RS	China	SI	198	35.4 ± 7.6			5 (0.03)	9 (5)	TVUS ± transabdominal US; ± gel stand-off
Harth 2023 [31]	RS	Germany	SI	160	33.0 ± 7.2		20 (0.13)		2 (1)	MRI 1.5T; T2 + T1 ± FS; moderate bladder filling; rectal & vaginal gel; whole pelvis ± upper urinary tract
Carfagna 2024 [32]	RS	Italy	SI	176	39.0	22.6	0 (0.00)	0 (0.00)	112 (64)	TVUS ± transabdominal US

*RS *retrospective study, *PS* prospective study, *SI* single centre, *MC* multicentre, *BMI* Body Mass Index, *MRI* magnetic resonance imaging, *TVUS* transvaginal ultrasound

**Table 2:** Characteristics of patients with bladder endometriosis

Study	Type	Country	Setting	*N*	Age (mean ± SD)	BMI (kg/m^2^) (mean ± SD)	Symptoms (%)	Endometriosis (%)	Imaging protocol
Bazot Darai 2004 [33]	PS	France	MC	195	34.2			16 (8)	MRI 1–1.5T; T2 + T1 ± FS; contrast; ± antispasmodic;
Bazot 2004 [34]	PS	France	SI	142	34.2		0 (0.0)	7 (5)	TVUS ± TAS; no bowel prep
Guerriero 2008 [35]	PS	Italy	SI	88	33 ± 5		0 (0.0)	4 (5)	TVUS; stand-off gel;
Hottat 2009 [36]	PS	Belgium	SI	41	33		5 (0.12)	2 (5)	MRI 3T; supine; T2 + T1 ± FS; rectal gel; antiperistaltic
Bazot 2009 [37]	RS	France	SI	92	31.8		3 (0.03)	3 (3)	TVUS-TRUS; no bowel prep. MRI 1.5T; T2 + T1 ± FS; contrast; antispasmodic
Savelli 2009 [23, 38]	RS	Italy	SI	490	33.1 ± 6		0 (0.00)	41 (8)	TVUS ± transabdominal; moderate bladder filling
Chamié 2009 [23]	PS	Brazil	SI	92	33		8 (0.09)	13 (14)	MRI: 1.5T; T2 + T1 FS; contrast; uro-MRI (selected cases)
Grasso 2010 [39]	PS	Italy	SI	26	33		0 (0.00)	6 (23)	3D TVUS; no bowel prep MRI 1.5T; T2 + T1 ± FS; bladder moderately filled; antispasmodic; ± contrast
Grasso 2010 [39]	PS	Italy	SI	33	33		0 (0.00)	6 (18)	
Bazot 2011 [40]	RS	France	SI	158	33.9 ± 6.9			8 (5)	MRI 1.5T; T2 + T1 ± FS; antispasmodic; contrast
Hudelist 2011 [8]	PS	UK, Austia	MC	129	32.2 ± 5.9		6 (0.05)	4 (3)	TVUS
Fratelli 2013 [41]	RS	Italy	SI	420	35	21	55 (0.13)	25 (6)	TVUS; no bowel prep
Vimercati 2012[24]	RS	Belgium	SI	90	34		16 (0.18)	6 (7)	TVUS; no bowel prep. MRI: 1.5T; T2 + T1 FS; contrast; antispasmodic
Holland 2013 [42]	PS	UK	MC	198	35 ± 7.1		0 (0.00)	5 (3)	TVUS; no bowel prep.
Kruger 2013 [43]	RS	Germany	SI	152	33.5 ± 6.1			21 (14)	MRI: 1.5T; T2 + T1 FS
Leon 2014 [44]	PS	Chile	SI	51	32.9		0 (0.00)	5 (10)	TVUS; bowel prep
Exacoustos 2014 [16]	PS	Italy	SI	104	35.6 ± 5.7		31 (0.3)	8 (8)	TVUS ± TRUS; no prep; partial bladder filling.
Di Paola 2015 [45]	RS	Italy	SI	115	36			12 (10)	MRI: 1.5T; T2 + T1 FS; rectal gel
Sillou 2015 [18]	RS	France	MC	35	33.5		33 (0.94)	13 (37)	MRI: 1.5T; T2 + T1 FS; ± contrast; uro-MRI
Tammaa 2015 [46]	PS	Austria	SI	65	30.2 ± 7.3		6 (0.09)	4 (6)	TVUS; no bowel prep
Brătilă 2016 [47]	PS	Romania	MC	193	32.4 ± 4		0 (0.00)	18 (9)	TVUS
Hudelist 2018 [25]	PS	Austria, UK	MC	207	33.9 ± 5.2		20 (0.09)	30 (15)	TVUS ± transabdominal US
Alborzi 2017 [26]	PS	Iran	SI	317	31 ± 5.4		0 (0.00)	4 (1)	TVUS/TRUS; bowel prep; partial bladder filling MRI: 1.5T; T2 + T1 FS; contrast; partial bladder; vaginal gel
Guerriero 2018 [20]	PS	Italy	SI	159	33		0 (0.00)	12 (8)	3D TVUS; bowel prep. MRI: 1.5T; T2 + T1 FS; contrast; antispasmodic
Hernández 2019 [19]	RS	Spain	SI	48	34 ± 6	22 ± 2.7	10 (0.21)	6 (13)	TVUS; bowel prep; partially filled bladder MRI: 1.5T; T2 + T1 FS; bowel prep; bladder & ureters; rectal & vaginal gel
Indrielle-Kelly 2020 [28]	PS	Czech Republic	SI	49	32.4 ± 5.3	23.6 ± 3.9	8 (0.16)	10 (20)	TVUS + transabdominal US; ± bowel prep; partial/moderate bladder filling MRI: 1.5–3T; T2 + T1 ± FS; ± bowel prep; moderate bladder filling; rectal & vaginal gel; whole pelvis ± upper urinary tract
Bielen 2020 [29]	PS	Belgium	SI	74	32			10 (14)	TVUS ± transabdominal US; ± bowel prep; partial/moderate bladder filling MRI: 1.5T; T2 + T1 ± FS; ± bowel prep; moderate bladder filling; rectal & vaginal gel; whole pelvis ± upper urinary tract
Yin 2020 [30]	RS	China	SI	198	35.4 ± 7.6			9 (5)	TVUS
Rousset 2021 [48]	RS	France	MC	37	31.2		32 (0.86)	31 (84)	MRI: 1.5T; T2 + T1 FS; vaginal gel
Barra 2021 [49]	PS	Italy	SI	194	34.9 ± 2.6	24.1 ± 2.1	7 (0.04)	34 (18)	TVUS ± 3D; full bladder;
Harth 2023 [50]	RS	Germany	SI	160	33 ± 7.2		20 (0.13)	6 (4)	MRI 1.5T; T2 + T1 ± FS; moderate bladder filling; rectal & vaginal gel; whole pelvis ± upper urinary tract
Piriyev 2025 [51]	RS	Germany	SI	42	34 ± 6.32	24.6 ± 7.2	32 (0.94)	37 (88)	TVUS + abdominal US for kidney involvement

*RS* retrospective study, *PS* prospective study, *SI* single centre, *MC* multicentre, *BMI* Body Mass Index

### Risk of bias assessment

A high risk of bias was observed in the patient selection domain, as the majority of studies recruited participants from tertiary referral centres, potentially enriching study populations with more severe disease.

In the index test domain, most studies were considered to be at low risk of bias, although some showed high or unclear risk, primarily due to lack of blinding to clinical information or variability in imaging protocols. For the reference standard, most studies demonstrated low risk of bias, while a smaller proportion showed unclear or high risk related to the lack of blinding of surgeons to preoperative imaging findings.

**Fig. 2:**
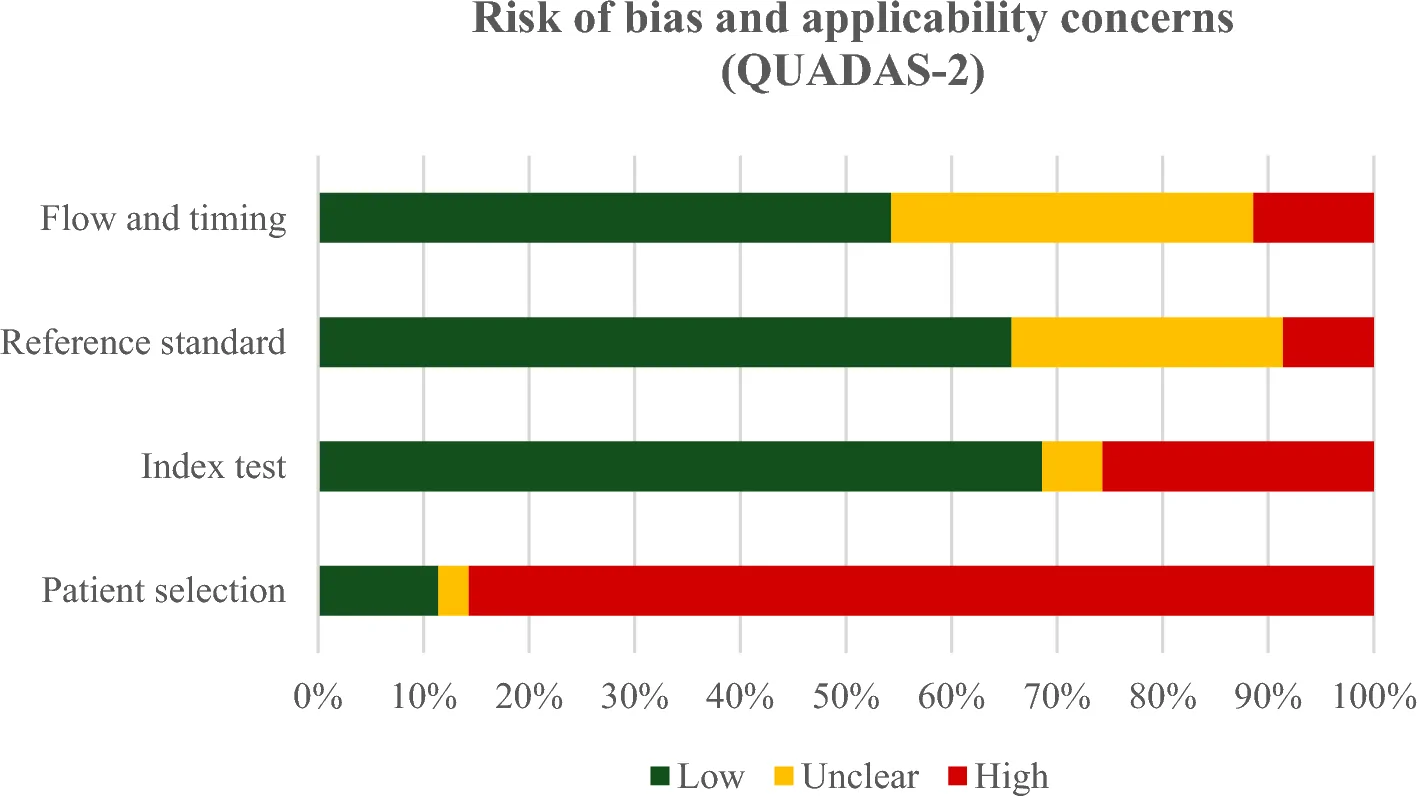
Risk of bias assessment using the QUADAS-2 tool

In the flow and timing domain, approximately half of the studies were assessed as low risk, whereas the remainder showed unclear or high risk, reflecting incomplete reporting of the interval between imaging and surgical confirmation or patient flow. Overall, applicability concerns were considered low across all domains (Fig. 2).

### Diagnostic accuracy for ureteral endometriosis

#### TVUS

**Fig. 3:**
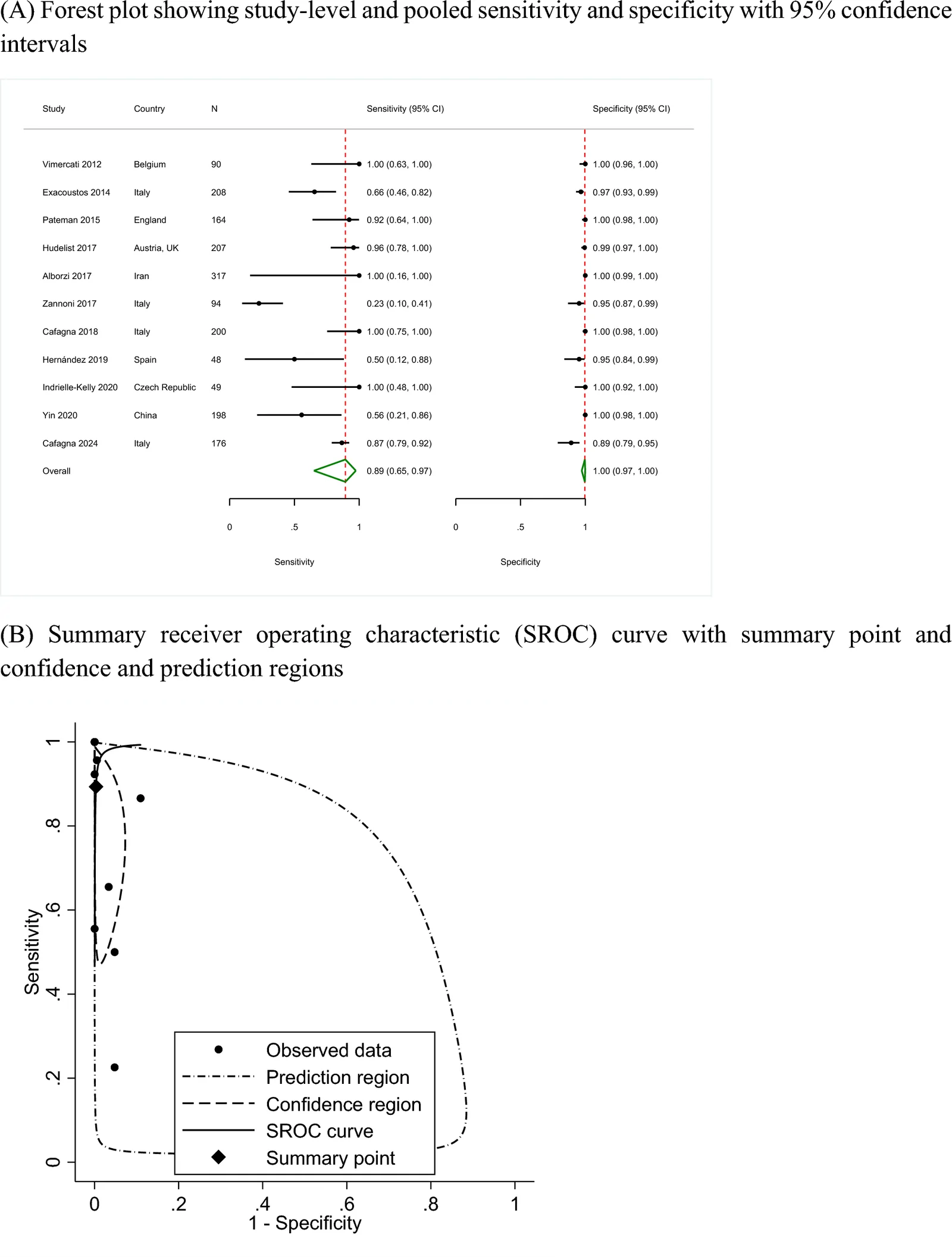
Diagnostic accuracy of TVUS for ureteral endometriosis. **A** Forest plot showing study-level and pooled sensitivity and specificity with 95% confidence intervals. **B** Summary receiver operating characteristic (SROC) curve with summary point and confidence and prediction regions

Eleven studies comprising 1,751 women evaluated the diagnostic performance of transvaginal ultrasound for ureteral endometriosis. The pooled sensitivity was 0.89 (95% CI 0.65–0.97) and the pooled specificity was 1.00 (95% CI 0.97–1.00) (Fig. 3). Sensitivity estimates varied across studies, ranging from 0.23 to 1.00, whereas specificity remained consistently high. The LR+ was extremely high due to specificity 1.00 and the LR- was 0.11. These findings indicate that TVUS demonstrates excellent specificity and high overall sensitivity for the detection of ureteral involvement.

#### MRI

**Fig. 4:**
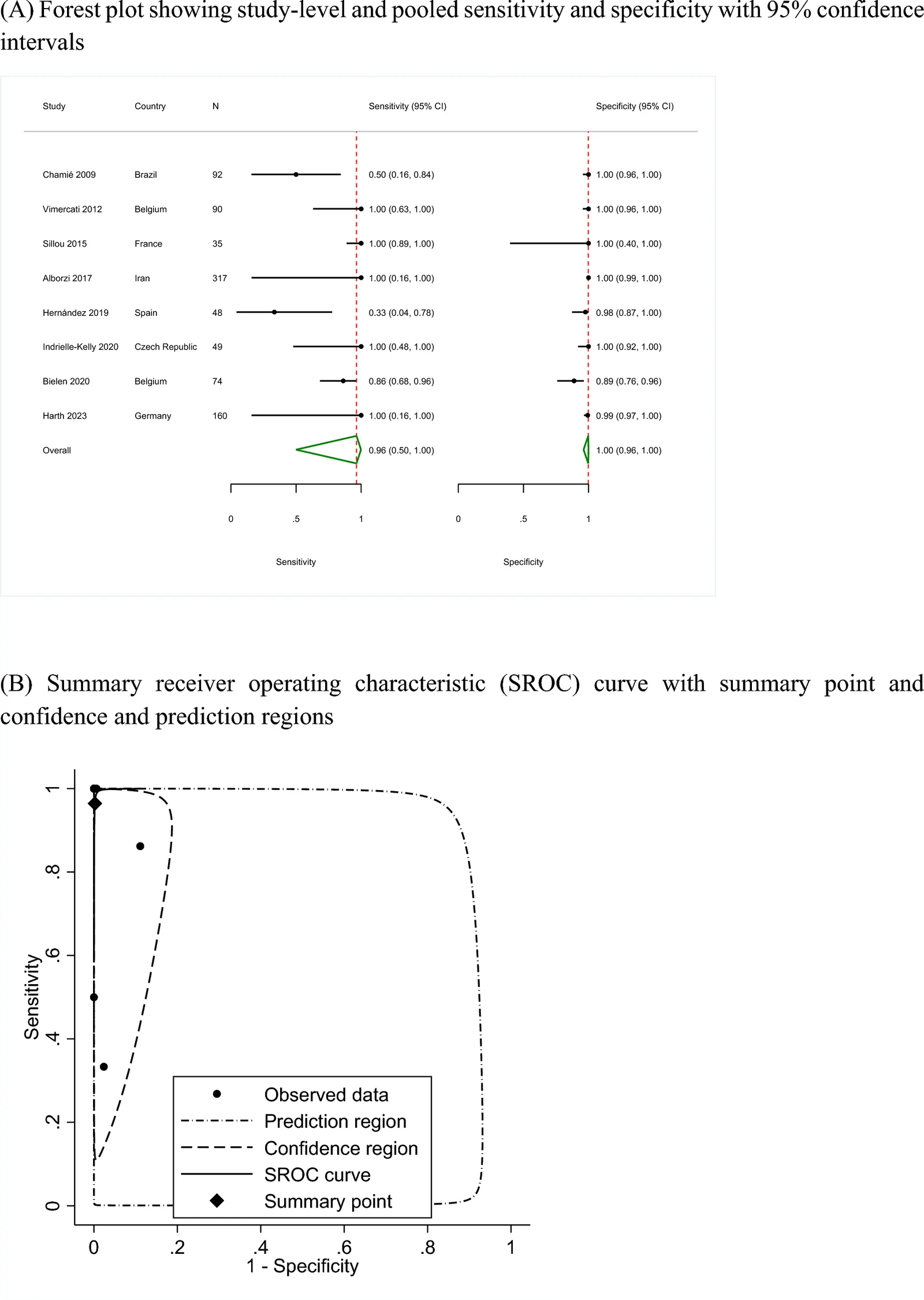
Diagnostic accuracy of MRI for ureteral endometriosis. **A** Forest plot showing study-level and pooled sensitivity and specificity with 95% confidence intervals. **B** Summary receiver operating characteristic (SROC) curve with summary point and confidence and prediction regions

Eight studies including approximately 865 women assessed the diagnostic performance of MRI for ureteral endometriosis. The pooled sensitivity was 0.96 (95% CI 0.50–1.00), and the pooled specificity was 1.00 (95% CI 0.96–1.00) (Fig. 4). The LR+ was extremely high due to specificity 1.00 and the LR− was 0.04. Although sensitivity varied across individual studies, MRI consistently demonstrated very high specificity and excellent diagnostic accuracy for ureteral disease.

### Diagnostic accuracy for bladder endometriosis

#### TVUS

**Fig. 5:**
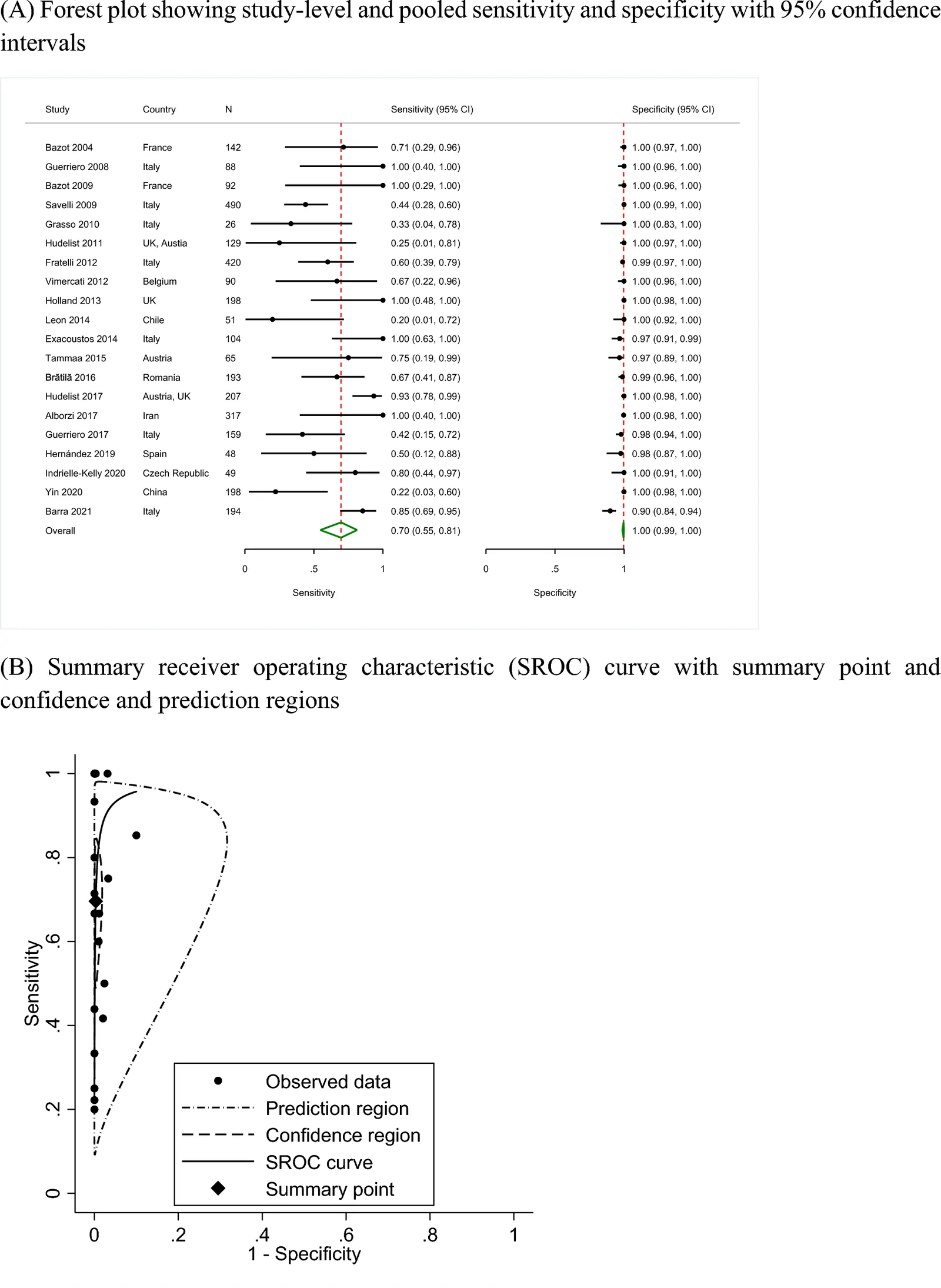
Diagnostic accuracy of TVUS for bladder endometriosis. **A** Forest plot showing study-level and pooled sensitivity and specificity with 95% confidence intervals. **B** Summary receiver operating characteristic (SROC) curve with summary point and confidence and prediction regions

Twenty-two studies involving approximately 3376 women assessed the diagnostic performance of transvaginal ultrasound for bladder endometriosis. The pooled sensitivity was 0.70 (95% CI 0.55–0.81), while the pooled specificity was 1.00 (95% CI 0.99–1.00) (Fig. 5). The LR+ was extremely high due to specificity 1.00 and the LR− was 0.30. Sensitivity estimates showed substantial variability across studies, ranging from 0.17 to 1.00, whereas specificity remained consistently high. These results suggest that TVUS is highly specific, but moderately sensitive for the detection of bladder involvement.

#### MRI

**Fig. 6:**
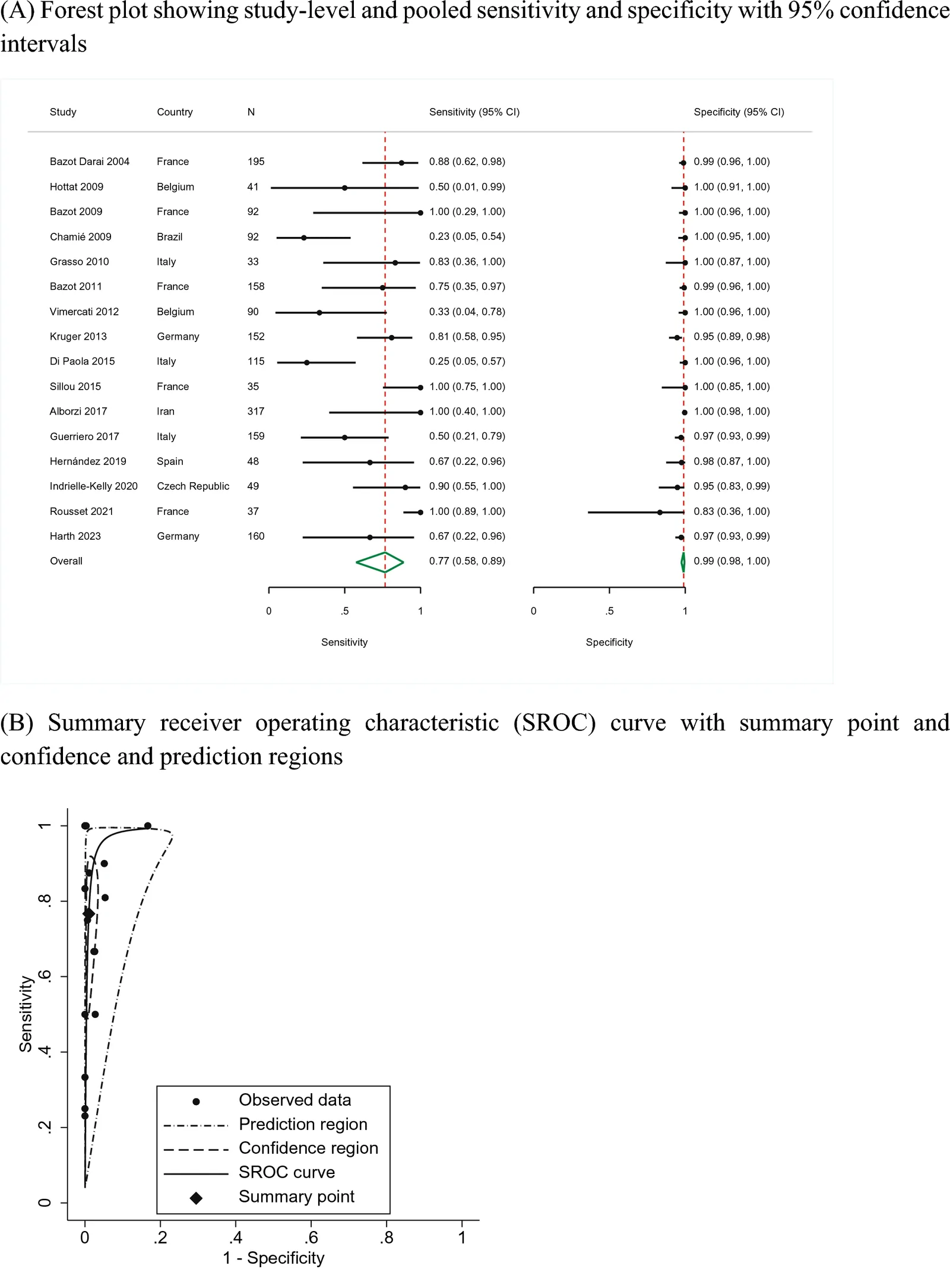
Diagnostic accuracy of MRI for bladder endometriosis. **A** Forest plot showing study-level and pooled sensitivity and specificity with 95% confidence intervals. **B** Summary receiver operating characteristic (SROC) curve with summary point and confidence and prediction regions

Fourteen studies including approximately 1773 women evaluated the diagnostic performance of MRI for bladder endometriosis. The pooled sensitivity was 0.77 (95% CI 0.58–0.89) and the pooled specificity was 0.99 (95% CI 0.98–1.00) (Fig. 6). The LR + was 77 and the LR- was 0.23. Sensitivity varied across studies, while specificity remained consistently high, indicating reliable exclusion of bladder involvement when MRI findings were negative.

### Sensitivity analysis including studies using both diagnostic methods

**Fig. 7:**
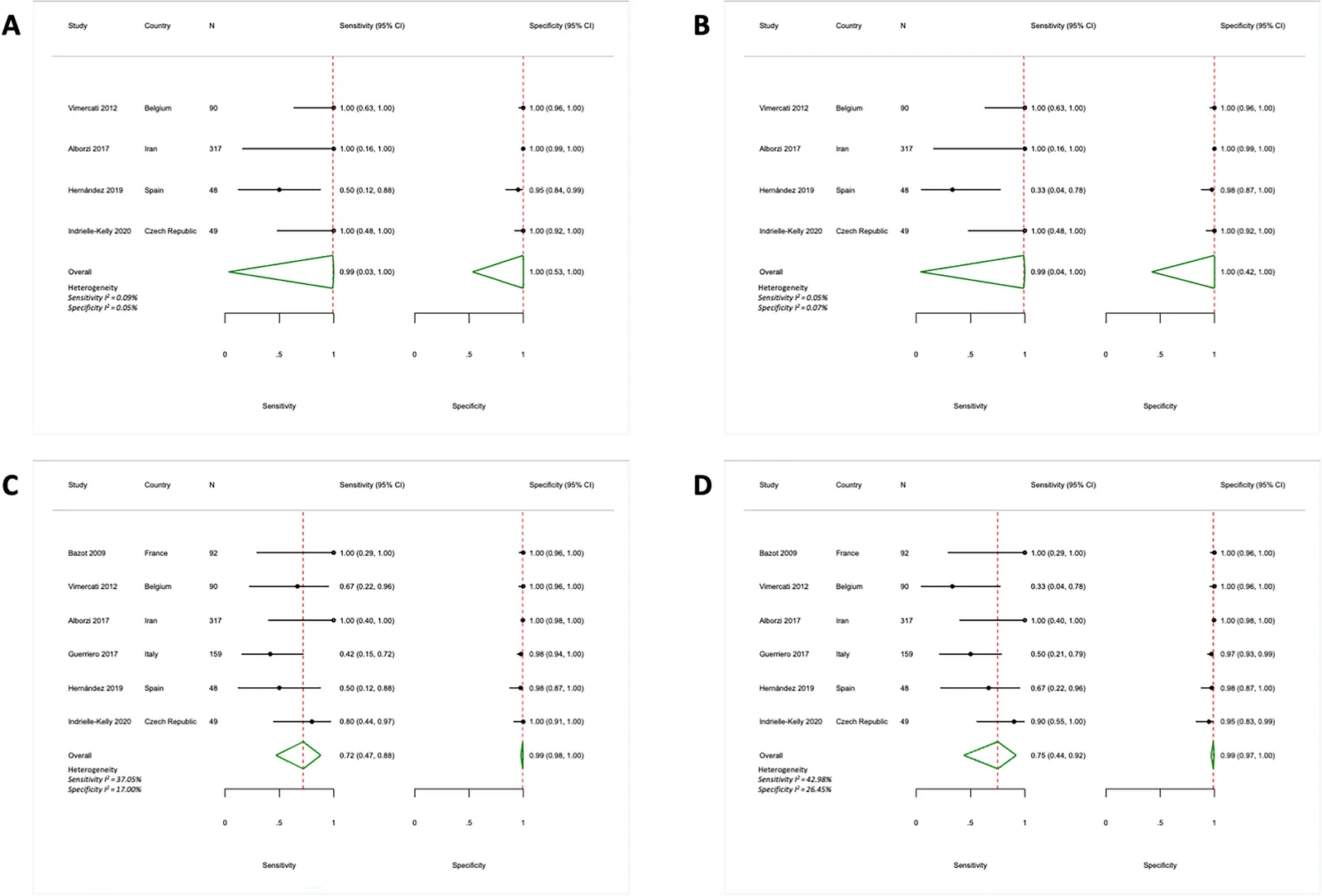
Diagnostic accuracy of **A** TVUS and **B** MRI for ureteral endometriosis and **C** TVUS and **D** MRI for bladder endometriosis in the sensitivity analysis including only studies that evaluated both diagnostic methods

Regarding the diagnostic accuracy for ureteral endometriosis, four studies comprising 504 women were included. The pooled sensitivity of TVUS was 0.99 (95% CI 0.03–1.00) and the pooled specificity was 1.00 (95% CI 0.53–1.00) (Fig. 7A). The pooled sensitivity of MRI was 0.99 (95% CI 0.04–1.00) and the pooled specificity was 1.00 (95% CI 0.42–1.00) (Fig. 7B). Regarding the diagnostic accuracy for bladder endometriosis, six studies comprising 755 women were included. The pooled sensitivity of TVUS was 0.72 (95% CI 0.47–0.88) and the pooled specificity was 0.99 (95% CI 0.98–1.00) (Fig. 7C). The pooled sensitivity of MRI was 0.75 (95% CI 0.44–0.92) and the pooled specificity was 0.99 (95% CI 0.97–1.00) (Fig. 7D).

## Discussion

This systematic review and meta-analysis provide an updated and comprehensive assessment of the diagnostic accuracy of TVUS and MRI for UTE, with separate analyses for ureteral and bladder involvement. The principal finding of this study is that both imaging modalities demonstrate consistently high specificity across anatomical sites, indicating a strong ability to correctly identify the absence of disease. In contrast, sensitivity varied according to imaging modality and anatomical location, with MRI showing numerically higher sensitivity for ureteral endometriosis; however, the overlap of the 95% confidence intervals suggests that a meaningful difference between the two modalities cannot be established.

However, the analysis by Tian et al. included studies published up to June 2020 and comprised a total of 23 studies. In contrast, the present review includes studies published up to December 2025 and incorporates 36 studies, reflecting the growing body of evidence on imaging-based diagnosis of urinary tract endometriosis in recent years.

The expanded dataset allowed a more comprehensive evaluation of diagnostic performance across anatomical sites. In particular, the number of studies assessing TVUS for ureteral endometriosis increased from 4 studies (1022 patients) in the analysis by Tian et al. to 11 studies (1751 patients) in the present review. Similarly, the evidence base for bladder endometriosis has nearly doubled, with 22 studies involving 3376 women included in our analysis compared with 13 studies (1736 patients) in the previous meta-analysis. This larger evidence base provides more robust pooled estimates and offers an updated synthesis of the available literature.

These findings are broadly consistent with previous literature evaluating imaging for UTE. A previous meta-analysis by Tian et al*.* evaluated the diagnostic performance of TVUS and MRI for bladder and ureteral endometriosis and reported high pooled specificity and moderate-to-high sensitivity, with better sensitivity for ureteral than bladder disease [15].

Differences in diagnostic performance between TVUS and MRI, particularly for ureteral involvement, likely reflect practical and technical characteristics of the two modalities rather than intrinsic superiority of one technique over the other. TVUS allows high-resolution, real-time assessment, high accessibility and has demonstrated good diagnostic performance in expert hands, but its accuracy is influenced by operator experience and adherence to structured examination protocols [16]. MRI, by contrast, provides a wider field of view and superior soft-tissue contrast, supporting comprehensive pelvic anatomical mapping and evaluation of associated posterior DIE [14]. In an early dedicated series, MRI enabled preoperative identification of ureteral endometriotic lesions and contributed to surgical planning, including the assessment of ureteral obstruction and hydronephrosis when urographic sequences were employed. Nevertheless, current evidence suggests that MRI has limited ability to reliably distinguish intrinsic from extrinsic ureteral involvement, highlighting an area where further methodological refinement is required [14].

Another factor that may influence the diagnostic performance of ultrasound for ureteral endometriosis is the completeness of urinary tract evaluation during imaging assessment. In clinical practice, systematic assessment of the upper urinary tract may reveal indirect signs, such as hydroureter or hydronephrosis, which can prompt a more targeted pelvic examination and facilitate identification of the causative ureteral lesion. Studies in which ultrasound examinations did not include systematic evaluation of the upper urinary tract may, therefore, underestimate the diagnostic performance of ultrasound for ureteral endometriosis [17]. Furthermore, intrinsic ureteral endometriosis is more frequently associated with ureteral obstruction and hydronephrosis, whereas extrinsic disease may be more difficult to detect in the absence of obstruction, which may partly contribute to heterogeneity in sensitivity estimates across studies [18].

For bladder endometriosis, both imaging modalities demonstrated high specificity, but only moderate sensitivity. This pattern is consistent with findings from individual diagnostic accuracy studies and indicates that imaging may not reliably identify all bladder lesions, particularly small or superficial disease. Consequently, negative imaging results should be interpreted with caution and considered alongside clinical presentation and other indicators of DIE [19].

From a clinical perspective, the consistently high specificity observed for both TVUS and MRI suggests that positive imaging findings are reliable and may support preoperative planning. In expert settings, TVUS has been widely used to assess pelvic endometriosis and allows evaluation of multiple pelvic compartments during a single examination, although diagnostic performance may vary according to operator expertise and examination protocols [8, 16, 20]. MRI provides complementary anatomical information and may be particularly useful when ureteral involvement is suspected or when broader pelvic mapping is required [14, 15]. Both TVUS and MRI demonstrated high diagnostic accuracy for urinary tract endometriosis. The high positive likelihood ratios and very low negative likelihood ratios observed in this analysis further support the strong rule-in and rule-out value of both imaging modalities, which is also reflected graphically by the SROC curves located near the upper left corner of the plots. A sensitivity analysis including only studies that evaluated both imaging modalities yielded similar estimates of diagnostic accuracy, supporting the robustness of the main findings, although confidence intervals were wider due to the smaller number of studies.

This study has several strengths, including the inclusion of recent evidence, stratification by anatomical site, and the use of a bivariate diagnostic accuracy model. However, several limitations must be acknowledged. Many included studies were conducted in tertiary referral centres with specialised expertise in endometriosis imaging and surgery. Such settings often include highly selected patient populations and a higher prevalence of advanced disease. Consequently, the diagnostic performance reported in these studies may be higher than that achievable in general clinical practice, where disease prevalence, operator experience and adherence to structured examination protocols may differ. Variability in imaging protocols, operator expertise, and reporting standards may have contributed to heterogeneity, particularly for TVUS. In addition, blinding of surgeons to preoperative imaging findings was often not reported, introducing potential bias in the reference standard. Differences in the unit of analysis for ureteral endometriosis (patient-based vs ureter-based) may also have influenced pooled estimates, as some studies analysed ureters individually rather than per patient. Intrinsic ureteral lesions, which infiltrate the ureteral wall, are often associated with hydronephrosis, whereas extrinsic lesions may be more challenging to detect on imaging [21]. Most studies included in this review did not specify this distinction, limiting the ability to evaluate its impact on diagnostic accuracy and representing a potential source of heterogeneity. In addition, standardized assessment of the anterior, lateral and posterior parametrium, as recommended by recent ISUOG guidelines, could improve the description of ureteral involvement and its relationship with surrounding tissues [22]. The lack of detailed reporting on ureteral subtypes may partly explain variability in sensitivity estimates across studies.

## Conclusion

In conclusion, both TVUS and MRI demonstrate excellent specificity for the detection of UTE. MRI shows slightly higher sensitivity for ureteral involvement, while both modalities exhibit moderate sensitivity for bladder disease. These findings support a complementary, site-specific imaging approach and emphasise the importance of specialist expertise and careful interpretation of negative imaging results in women with suspected urinary tract endometriosis. Future prospective studies using standardised imaging protocols and clearly defined reference standards are warranted to further refine diagnostic pathways for urinary tract endometriosis.

## Supplementary Information

Below is the link to the electronic supplementary material.

Supplementary file1 (PDF 766 KB)

## Data Availability

All data supporting the findings of this study are available within the paper and its Supplementary Information.
